# Myocardial work in light-chain and transthyretin-related amyloidosis with preserved ejection fraction: a cardiovascular magnetic resonance study

**DOI:** 10.1186/s13244-026-02408-z

**Published:** 2026-10-09

**Authors:** Shichu Liang, Jing Chen, Qiao Deng, Danni Li, Ke Wan, Keying Bi, Jiayu Sun, Qing Zhang, Yuchi Han, Yucheng Chen

**Affiliations:** 1 Department of Cardiology, West China Hospital, Sichuan University, Chengdu, China; 2 Department of Geriatrics and National Clinical Research Center for Geriatrics, West China Hospital, Sichuan University, Chengdu, China; 3 Department of Radiology, West China Hospital, Sichuan University, Chengdu, China; 4 Center of Rare Diseases, West China Hospital, Sichuan University, Chengdu, China; 5 Cardiovascular Division, Wexner Medical Center, The Ohio State University, Columbus, OH USA; 6 Cardiac Imaging and Target Therapy Lab, West China Hospital, Sichuan University, Chengdu, China; 7 Frontiers Medical Center, Tianfu Jincheng Laboratory, Chengdu, China

**Keywords:** Cardiomyopathy, Amyloidosis, Magnetic resonance imaging, Differential diagnosis

## Abstract

**Objectives:**

To investigate pressure–volume loop (PV-loop) changes by cardiovascular magnetic resonance (CMR) imaging in cardiac amyloidosis (CA) patients with preserved left ventricular ejection fraction (LVEF), and to assess PV-loop parameters’ potential as subtype differential diagnostic markers.

**Materials and methods:**

Between May 2017 and September 2023, 100 consecutive confirmed CA patients (14 transthyretin-related [ATTR-CA] and 86 light-chain [AL-CA]) and 40 age- and sex-matched healthy controls (HCs) were prospectively enrolled. PV-loop parameters were derived from CMR cine images and brachial blood pressure. Clinical and CMR parameters were compared between CA patients and HCs, as well as between CA subtypes. The correlations between PV-loop parameters and clinical and imaging parameters were assessed. Furthermore, the differential diagnostic performance of these PV-loop parameters was evaluated using the area under the receiver operating characteristic curve (ROC-AUC). Bootstrapping was performed 2000 times for internal validation.

**Results:**

Compared to HCs, CA patients showed significantly lower stroke work index, potential energy index (PEI), and work efficiency (all *p* < 0.001). AL-CA patients had higher N-terminal pro-B-type natriuretic peptide (NT-proBNP) levels (2358.00 [IQR: 903.25, 5559.00] vs 810.00 [IQR: 269.00, 1136.01] pg/mL, *p* = 0.002) and lower PEI (0.13 [IQR: 0.07, 0.15] vs 0.15 [IQR: 0.13, 0.19] J/m², *p* = 0.025) than ATTR-CA patients. NT-proBNP (β = −1.94, OR = 0.14, 95% CI: 0.03–0.64, *p* = 0.016) and PEI (β = 0.20, OR = 1.23, 95% CI: 1.07–1.44, *p* = 0.007) were independent predictors for AL-CA vs ATTR-CA differentiation, and combining these two parameters enhanced the diagnostic performance (bootstrapping AUC 0.847).

**Conclusion:**

By providing pathophysiological insights into the distinctions between AL-CA and ATTR-CA, PV-loop parameters have the potential to serve as a complementary, contrast-free approach for differential diagnosis.

**Key Points:**

***Question*** PV-loop changes by CMR in CA patients with preserved LVEF remain unknown.

***Findings*** Light-chain CA patients with preserved LVEF had higher NT-proBNP levels and lower PEI than transthyretin-related CA patients.

***Critical relevance statement*** The CMR-derived PV-loop parameters revealed pathophysiological differences between the two subtypes of CA, suggesting potential differential diagnostic value.

**Graphical Abstract:**

**Figure:**
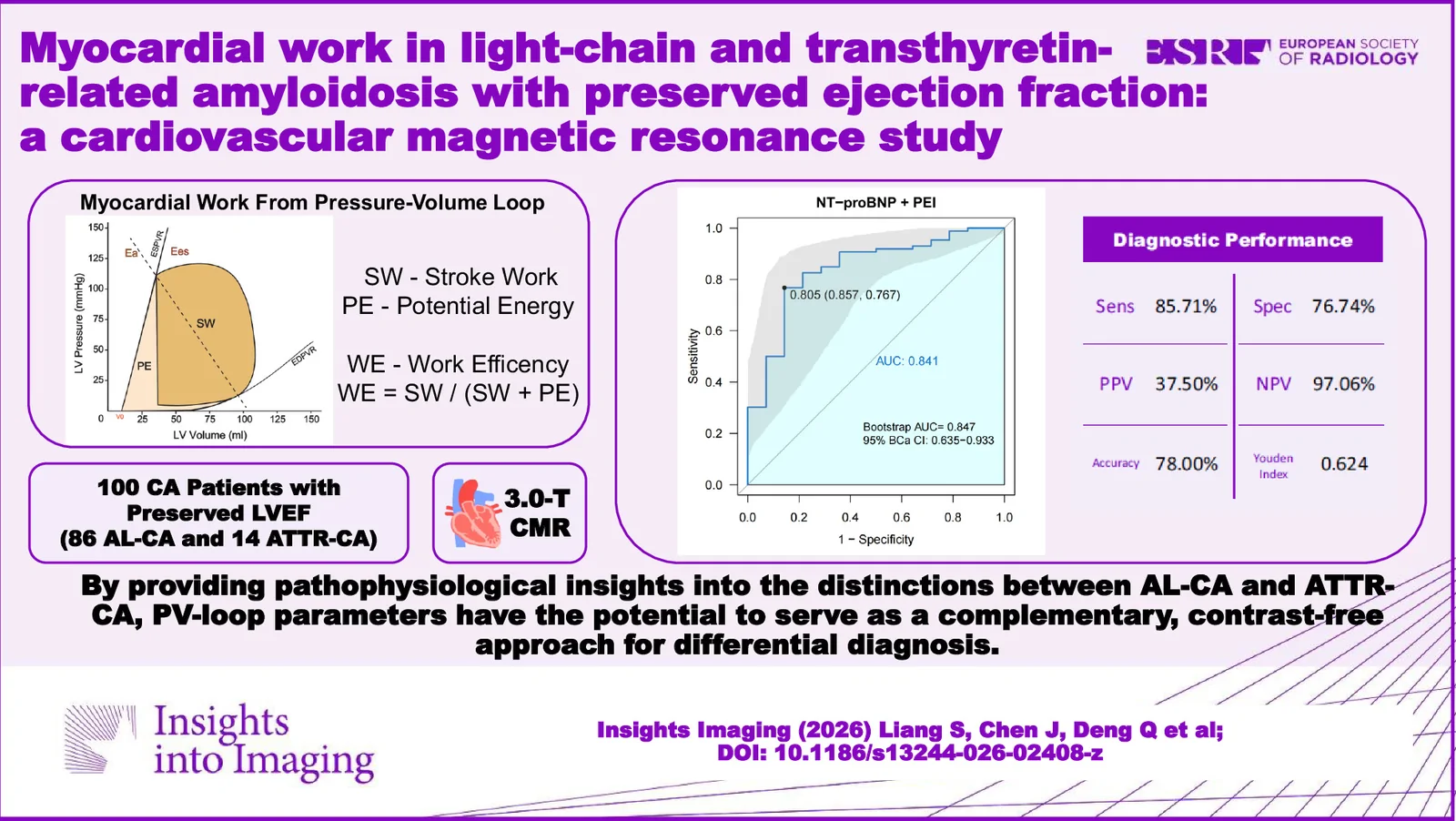

## Introduction

Cardiac amyloidosis (CA) is a progressive infiltrative cardiomyopathy with life-threatening consequences resulting from the deposition of amyloid fibrils within myocardial tissue [1]. The disease spectrum is dominated by two principal subtypes: immunoglobulin light-chain (AL) and transthyretin-related (ATTR) amyloidosis, which collectively constitute approximately 98% of clinically diagnosed cases [2]. Despite growing recognition, diagnostic delays remain pervasive in clinical practice. Recent registry data indicated a median diagnostic latency exceeding 12 months from symptom onset for both subtypes [3, 4]. By this time, most patients have developed advanced cardiac involvement with substantially limited therapeutic windows. The natural history varies markedly between subtypes: Untreated light-chain cardiac amyloidosis (AL-CA) exhibits a median survival of < 6 months [3], whereas untreated transthyretin-related cardiac amyloidosis (ATTR-CA) demonstrates a more protracted median survival of 3–5 years [4]. Given these disparities, early detection and accurate phenotyping of CA are critical prerequisites for initiating subtype-specific therapy and improving patient survival.

Although endomyocardial biopsy with histopathological confirmation remains the diagnostic gold standard [5], its invasive nature has constrained its widespread clinical adoption. Contemporary diagnostic algorithms increasingly incorporate advanced noninvasive imaging modalities, which now occupy a central diagnostic role. Established techniques include echocardiography, cardiovascular magnetic resonance (CMR), and ^99m^Tc-pyrophosphate (PYP) scintigraphy [6]. Among these, CMR has emerged as the preeminent imaging modality for CA characterization, offering unparalleled soft-tissue resolution, sophisticated tissue characterization capabilities (particularly through late gadolinium enhancement [LGE] and T1 mapping), and the absence of ionizing radiation [7, 8]. Left ventricular (LV) global longitudinal strain (GLS) is recognized as a sensitive marker of subclinical myocardial dysfunction [9], demonstrating potential utility in CA differential diagnosis using echocardiography or CMR [10, 11]. However, left ventricular global longitudinal strain (LV-GLS) interpretation in CA is complicated by its inherent load dependence, a particular challenge in CA populations where volume overload, diuretic use, and hypotension frequently alter loading conditions [12, 13].

Pressure–volume loop (PV-loop) analysis provides a comprehensive hemodynamic assessment by simultaneously measuring LV volume and pressure. While conventionally requiring invasive catheterization, recent methodological advances have enabled entirely noninvasive PV-loop derivation using CMR data [14, 15], allowing for a quantitative assessment of cardiac thermodynamic efficiency without procedural risks. Although echocardiography-derived pressure-strain loop (PSL) analysis has demonstrated diagnostic promise in CA [16], the clinical implications of CMR-based PV-loop analysis remain unexplored. Therefore, this study aimed to (1) systematically evaluate PV-loop parameters in patients with CA and their associations with disease activity and amyloid burden; (2) explore the discriminant capacity of PV-loop analysis for distinguishing between AL and ATTR amyloidosis subtypes.

## Materials and methods

The Institutional Review Board of West China Hospital, Sichuan University approved the study (ethics approval number: 20190381), and it was registered with the Chinese Clinical Trial Registry (ChiCTR1900024764). Written informed consent was obtained from all participants before enrollment.

### Study population and clinical evaluation

This single-center, prospective cohort study enrolled 14 consecutive patients with ATTR-CA and 86 patients with AL-CA who underwent contrast-enhanced CMR imaging at West China Hospital, Sichuan University (Chengdu, China), from May 2017 to September 2023. All patients were diagnosed following the current international guidelines and had either histological confirmation of amyloid deposition on cardiac or extracardiac biopsy or previously established non-biopsy diagnostic criteria (Supplementary Materials) [17–19]. Additionally, all patients had preserved left ventricular ejection fraction (LVEF), defined as ≥ 50% [20]. Cardiac involvement was defined by the presence of any of the following: (1) a positive endomyocardial biopsy; (2) LV wall thickness > 12 mm on echocardiography in the absence of other identifiable causes; (3) a typical diffuse subendocardial or transmural LGE pattern on CMR imaging; or (4) an extracellular volume (ECV) fraction ≥ 40% [18, 21]. Patients without cardiac involvement, such as those with isolated renal AL amyloidosis or asymptomatic TTR variant carriers, were excluded in this study. Details of the included patients are presented in Supplementary Materials.

All 100 CA patients underwent laboratory assessments, including serum creatinine (SCr), N-terminal pro-B-type natriuretic peptide (NT-proBNP), and cardiac troponin T (cTnT) measurements. Cardiac functional classification was determined according to the New York Heart Association (NYHA) criteria [22]. Forty age- and sex-matched healthy controls (HCs) were selected from our healthy volunteer database [23]. Seated blood pressure was measured following a 5-min rest period prior to CMR examination [24, 25]. Clinical data were collected by two independent investigators (S. Liang and J. Chen).

### CMR imaging protocol

CMR examinations were performed using a 3.0-T scanner (MAGNETOM Trio or Skyra; Siemens Healthcare, Erlangen, Germany) with a 32-channel phased-array body coil. All participants underwent CMR in the supine position. Steady-state free precession (SSFP) cine images were acquired in consecutive short-axis (SAX) slices covering the entire LV and three long-axis views (2-, 3-, and 4-chamber). T1 mapping was performed using a Modified Look-Locker Inversion-recovery sequence before and 10–15 min after contrast injection at the basal-, mid-, and apical-ventricular SAX slice level. T2 mapping was performed using the T2-prepared single-shot bSSFP technique before gadolinium administration on SAX views matching those for T1 mapping. LGE imaging was performed approximately 15 min after intravenous administration of a gadolinium-based contrast agent, with consecutive SAX slices covering the LV. The detailed acquisition parameters are provided in Supplementary Materials.

### CMR post-processing and analysis

Biventricular volumes, ejection fraction, and LV mass were quantified using the QMASS software package (Version 8.1; Medis Medical Imaging Systems, Leiden, The Netherlands). The biventricular end-diastolic and end-systolic volumes and LV mass were indexed to body surface area (BSA) for normalization [26].

Patients with CA were divided into three subgroups based on the LGE extent observed 10–15 min post-contrast injection [21]: (a) No LGE group (no LGE in any section); (b) subendocardial LGE group (diffuse subendocardial LGE with transmurality < 50% in any SAX section); (c) transmural LGE group (diffuse LGE with transmurality ≥ 50% in at least one SAX section). Two independent readers (S. Liang and K. Wan), blinded to clinical data, assessed LGE patterns. For discrepancies, a third expert (Y. Chen, with 10 years of experience) arbitrated. Native T1, ECV, and T2 analyses are provided in the Supplementary Materials.

### LV strain and PV-loop analysis

Strain and PV-loop analysis was performed using the QStrain package (Version 4.2; Medis Medical Imaging Systems, Leiden, the Netherlands). The LV global circumferential and radial strains (LV-GCS, LV-GRS) were derived from three specific slices of the SAX stack: basal, mid-ventricular, and apical levels. The basal slice was chosen for its visibility of a complete circle of myocardium throughout the entire cardiac cycle, whereas the apex was selected based on the retention of LV cavity visibility during systole. Subsequently, LV-GLS was manually calculated from the three long-axis (2-, 3-, and 4-chamber) cine images by two independent readers (S. Liang and Q. Deng). After inputting brachial systolic and diastolic pressures, the PV-loop was reconstructed, and PV-loop variables were defined based on the generated loops [27].

**Fig. 1:**
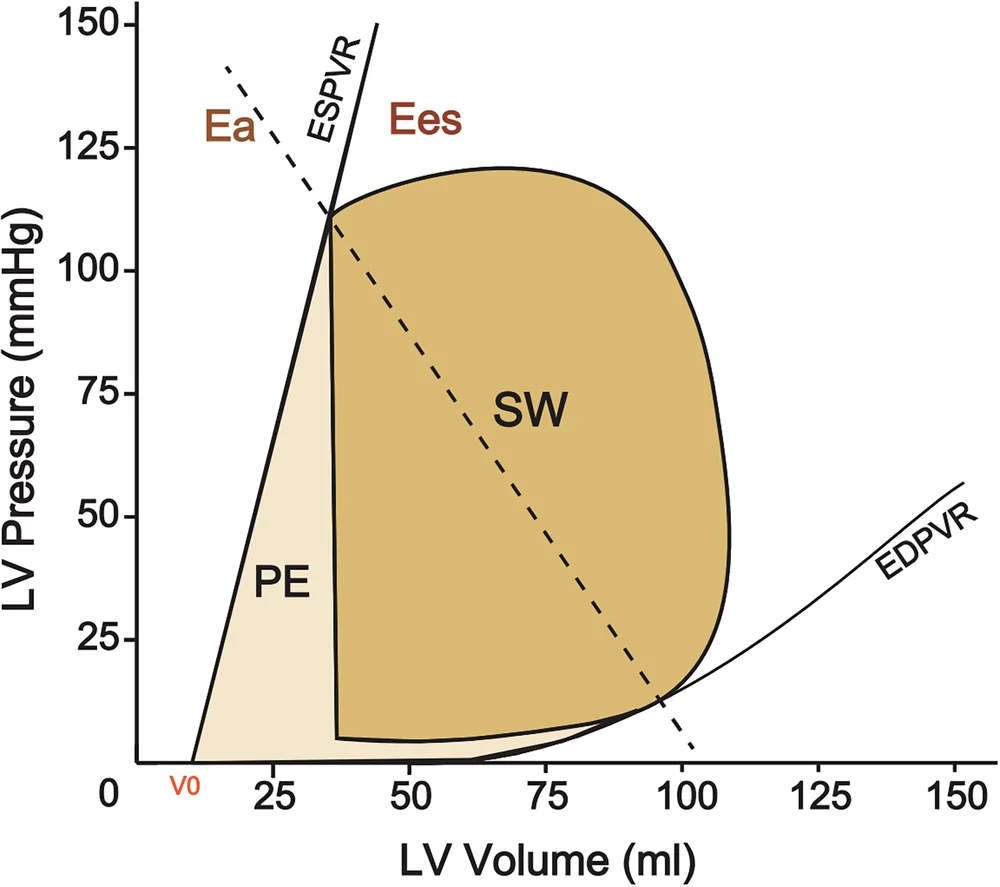
Illustration of the left ventricular PV-loop. Ea, arterial elastance; Ees, end-systolic elastance; EDPVR, end-diastolic pressure–volume relationship; ESPVR, end-systolic pressure–volume relationship; LV, left ventricular; SW, stroke work; PE, potential energy

The end-systolic pressure–volume relationship (ESPVR) was derived by plotting end-systolic pressure–volume points across consecutive beats, forming a linear curve intersecting the x-axis at end-systolic volume (V0). The slope of this curve quantifies end-systolic elastance (Ees), a measure of myocardial contractility. The negative slope from the ESPVR point to the end-diastolic volume on the PV-loop represents effective arterial elastance (Ea), assessing arterial load. Stroke work (SW) was defined as the area within the PV-loop, whereas potential energy (PE) was defined as the area enclosed by the Ees line, the x-axis (pressure = 0), and the left side of the PV-loop, representing the energy required for the left ventricle to eject the stroke volume. Pressure–volume area (PVA) was defined as SW + PE. Work efficiency (WE) was calculated as SW/ PVA, and the ventricular-arterial coupling (VAC) index was calculated as Ea/Ees. PVA, SW, and PE were indexed to BSA to facilitate comparisons among participants with different body sizes, expressed as stroke work index (SWI) and potential energy index (PEI) [27]. A representative PV-loop is illustrated in Fig. 1.

### Statistical analysis

Statistical analyses were performed using R software (version 4.2.2). Data normality was assessed using the Kolmogorov-Smirnov test. Normally distributed continuous variables are presented as mean ± standard deviation, and were compared between groups using a *t*-test. Non-normally distributed variables are reported as median (interquartile range, IQR) and were compared using the Mann–Whitney *U*-test. Categorical variables were expressed as percentages and analyzed using the chi-square test or Fisher’s exact test, as appropriate. Correlation coefficient heatmaps were generated using Pearson correlation coefficients (*r*) based on distance measurements. The correlation coefficients (*r*-values) were classified as follows: Weak (0 < *r* < 0.2), mild (0.2 ≤ *r* < 0.4), moderate (0.4 ≤ *r* < 0.6), moderately strong (0.6 ≤ *r* < 0.8), and strong (0.8 ≤ *r* < 1.0). Univariate logistic regression analysis was used to evaluate the clinical and CMR parameters for differentiating AL-CA from ATTR-CA. Parameters that were significantly associated with this differentiation in the univariate analysis (*p* < 0.1) were subsequently included in the multivariable logistic regression analysis. Receiver operating characteristic (ROC) analysis was applied to identify the optimal parameter for differentiating AL-CA from ATTR-CA, with cutoff values determined using the maximum Youden’s Method [28]. Diagnostic accuracy was evaluated and internally validated using bootstrapping with 2000 repetitions to mitigate optimistic bias using the boot package. For reproducibility analysis, 20 patients were randomly selected. Two independent readers (S. Liang and D. Li), blinded to all clinical information, performed the analysis to assess inter-observer agreement. For intra-observer agreement, one reader repeated the analysis after a two-month washout period, blinded to prior results. Both inter- and intra-observer agreement were quantified using the intraclass correlation coefficient (ICC) and Bland-Altman analysis. Additionally, the reproducibility of PV loop parameters derived from single (4-chamber) vs multi-chamber (2-, 3-, 4-chamber) long-axis cine reconstructions was evaluated. For all analyses, two-sided *p* values were reported, with statistical significance set at *p* < 0.05.

## Results

### Demographic and baseline clinical characteristics

The clinical characteristics of patients with CA and HCs are summarized in Table 1. Specifically, patients with CA had lower systolic blood pressure (113.09 ± 19.83 vs 122.12 ± 11.70 mmHg, *p* = 0.001) and higher heart rate (79.56 ± 14.28 vs 75.28 ± 9.09 b.p.m., *p* = 0.037) than HCs. Comparisons of the clinical characteristics of patients with ATTR-CA and AL-CA are summarized in Table 2. Among patients with ATTR-CA and AL-CA, the prevalence of cardiovascular comorbidities and NYHA stages was similar. Laboratory analyses revealed that patients with AL-CA had higher NT-proBNP levels (2358.00 [IQR: 903.25, 5559.00] vs 810.00 [IQR: 269.00, 1136.01] pg/mL, *p* = 0.002) and cTnT levels (56.80 [IQR: 26.48, 100.55] vs 30.35 [IQR: 26.40, 34.92] ng/L, *p* = 0.014) than patients with ATTR-CA, whereas SCr levels were comparable between the two groups.

**Table 1:** Clinical characteristics and cardiac magnetic resonance parameters of patients with CA and HCs

	HCs (*n* = 40)	CA (*n* = 100)	*p* value
Demographic characters			
Age (years)	59.50 (46.75, 64.00)	59.00 (52.00, 66.00)	0.262
Males, *n* (%)	23 (57.50)	62 (62.00)	0.763
SBP (mmHg)	122.12 ± 11.70	113.09 ± 19.83	**0.001**
DBP (mmHg)	73.00 (70.00, 80.25)	71.50 (63.75, 79.50)	0.121
Heart rate (b.p.m.)	75.28 ± 9.09	79.56 ± 14.28	**0.037**
BMI (kg/m^2^)	22.83 ± 2.91	22.68 ± 3.28	0.78
BSA (m^2^)	1.58 ± 0.19	1.60 ± 0.16	0.558
CMR parameters			
LV wall thickness (mm)	8.47 (7.57, 9.39)	16.00 (14.00, 18.00)	**< 0.001**
LVEDVi (mL/m^2^)	74.09 (68.09, 78.43)	70.20 (62.54, 78.55)	0.087
LVESVi (mL/m^2^)	27.44 ± 6.59	28.88 ± 7.30	0.262
LVMassi (g/m^2^)	45.56 (40.10, 52.58)	77.78 (64.63, 95.51)	**< 0.001**
LVEF (%)	63.65 (57.88, 66.98)	58.32 (54.22, 64.09)	**< 0.001**
RVEDVi (mL/m^2^)	69.12 ± 15.11	63.87 ± 18.16	0.084
RVESVi (mL/m^2^)	29.48 (24.96, 34.14)	25.86 (21.40, 34.24)	0.187
RVEF (%)	55.53 (50.18, 61.79)	56.25 (48.53, 61.40)	0.985
LV-GLS (%)	−22.34 ± 3.29	−14.59 ± 4.75	**< 0.001**
LV-GCS (%)	−22.92 (−24.61, −20.34)	−21.36 (−23.66, −18.73)	0.05
LV-GRS (%)	71.45 (59.00, 80.97)	60.13 (44.91, 75.61)	**0.026**
Global native T1 (ms)	1204.60 (1177.22, 1235.35)	1424.04 (1382.99, 1465.16)	**< 0.001**
Global ECV (%)	26.92 (25.70, 28.98)	47.17 (41.32, 51.47)	**< 0.001**
Global Native T2 (ms)	40.90 (39.77, 42.36)	45.35 (42.25, 47.21)	**< 0.001**
PV-loop analysis			
End-systolic Elastance (Ees, mmHg/mL)	3.60 (2.60, 3.92)	3.30 (2.80, 4.60)	0.834
Arterial Elastance (Ea, mmHg/mL)	1.45 (1.30, 1.80)	1.75 (1.40, 2.32)	**0.007**
Ventricular-Arterial Coupling index (%)	45.03 (36.72, 55.67)	55.17 (39.87, 64.21)	**0.014**
Stroke Work (J)	0.95 (0.80, 1.20)	0.70 (0.50, 0.90)	**< 0.001**
Stroke Work Index (J/m^2^)	0.62 ± 0.12	0.46 ± 0.20	**< 0.001**
Potential Energy (J)	0.30 (0.20, 0.30)	0.20 (0.10, 0.30)	**< 0.001**
Potential Energy Index (J/m^2^)	0.17 (0.14, 0.20)	0.13 (0.07, 0.16)	**< 0.001**
Pressure–volume area (J)	1.25 (1.00, 1.50)	0.90 (0.70, 1.10)	**< 0.001**
Pressure–volume area index (J/m^2^)	0.79 ± 0.13	0.59 ± 0.23	**< 0.001**
Work Efficiency (%)	80.90 (76.92, 83.35)	76.55 (73.05, 81.90)	**0.005**

*L/RVEF* left/right ventricular ejection fraction, *LVEDVi/RVEDVi* left/right ventricular end-diastolic volume index, *LVESVi/RVESVi* left/right end-systolic volume index, *LV-GCS* left ventricular global circumferential strain, *LV-GLS* left ventricular global longitudinal strain, *LV-GRS* left ventricular global radial strain, *LVMassi* left ventricular mass index, *SBP* systolic blood pressure, *DBP* diastolic blood pressure, *BMI* body mass index, *BSA* body surface area

Bold values indicate statistical significance *p* < 0.05

**Table 2:** Clinical characteristics in patients with light-chain and transthyretin-related amyloidosis

	Total CA patients (*n* = 100)	AL-CA (*n* = 86)	ATTR-CA (*n* = 14)	*p* value
Demographic characters				
Age (years)	59.00 (52.00, 66.00)	59.50 (53.00, 66.00)	55.50 (47.50, 68.00)	0.39
Males, *n* (%)	62 (62.00)	52 (60.47)	10 (71.43)	0.626
SBP (mmHg)	113.09 ± 19.83	112.27 ± 19.34	118.14 ± 22.77	0.374
DBP (mmHg)	71.50 (63.75, 79.50)	70.00 (63.25, 79.00)	74.00 (69.50, 83.00)	0.453
Heart rate (b.p.m.)	79.56 ± 14.28	80.53 ± 14.21	73.57 ± 13.67	0.096
BMI (kg/m^2^)	22.68 ± 3.28	23.05 ± 3.05	20.39 ± 3.86	**0.026**
BSA (m^2^)	1.60 ± 0.16	1.60 ± 0.16	1.57 ± 0.14	0.471
Cardiovascular comorbidities				
Smoking, *n* (%)	27 (27.00)	25 (29.07)	2 (14.29)	0.34
Drinking, *n* (%)	18 (18.00)	17 (19.77)	1 (7.14)	0.455
Hypertension, *n* (%)	19 (19.00)	18 (20.93)	1 (7.14)	0.296
Diabetes mellitus, *n* (%)	6 (6.00)	5 (5.81)	1 (7.14)	1
NYHA functional class				0.553
I, *n* (%)	19 (19.00)	16 (18.60)	3 (21.43)	…
II, *n* (%)	35 (35.00)	28 (32.56)	7 (50.00)	…
III, *n* (%)	41 (41.00)	37 (43.02)	4 (28.57)	…
IV, *n* (%)	5 (5.00)	5 (5.81)	0 (0.00)	…
Laboratory examination				
SCr (umol/L)	81.50 (66.00, 113.25)	83.50 (66.00, 127.75)	75.62 (64.50, 82.50)	0.1
cTnT (ng/L)	50.25 (26.32, 90.42)	56.80 (26.48, 100.55)	30.35 (26.40, 34.92)	**0.014**
NT-proBNP (pg/mL)	1806.00 (724.00, 5044.50)	2358.00 (903.25, 5559)	810.00 (269.00, 1136.01)	**0.002**

*BMI* body mass index, *BSA* body surface area, *cTnT* cardiac troponin T, *DBP* diastolic blood pressure, *dFLC* serum free light-chain difference, *NT-proBNP* N-terminal pro-B-type natriuretic peptide, *SBP* systolic blood pressure, *SCr* serum creatinine

Bold values indicate statistical significance *p* < 0.05

### CMR and myocardial work characteristics between CA patients and HCs

The CMR characteristics of patients with CA and HCs are summarized in Table 1. Compared with HCs, patients with CA exhibited significantly higher LV wall thickness (16.00 [IQR: 14.00, 18.00] vs 8.47 [IQR: 7.57, 9.39] mm, *p* < 0.001) and LV mass index (LVMassi, 77.78 [IQR: 64.63, 95.51] vs 45.56 [IQR: 40.10, 52.58] g/m^2^, *p* < 0.001). Moreover, despite preserved LVEF, the LVEF (58.32 [IQR: 54.22, 64.09] vs 63.65 [IQR: 57.88, 66.98] %, *p* < 0.001), LV-GLS (−14.59 ± 4.75 vs −22.34 ± 3.29%, *p* < 0.001), and LV-GRS (60.13 [IQR: 44.91, 75.61] vs 71.45 [IQR: 59.00, 80.97]%, *p* = 0.026) of patients with CA were still lower than those in HCs, indicating subclinical myocardial impairment.

Additionally, the native T1 value (1424.04 [IQR: 1382.99, 1465.16] vs 1204.60 [IQR: 1177.22, 1235.35] ms, *p* < 0.001), ECV (47.17 [IQR: 41.32, 51.47] vs 26.92 [IQR: 25.7, 28.98] %, *p* < 0.001), and T2 value (45.35 [IQR: 42.25, 47.21] vs 40.90 [IQR: 39.77, 42.36] ms, *p* < 0.001) were also higher in patients with CA than in HCs, indicating the fibrosis presence, amyloid deposition, and myocardial edema in patients with CA.

In terms of myocardial work, patients with CA exhibited both lower SWI (0.46 ± 0.20 vs 0.62 ± 0.12 J/m^2^, *p* < 0.001) and PEI (0.13 [IQR: 0.07, 0.16] vs 0.17 [IQR: 0.14, 0.20] J/m^2^, *p* < 0.001), resulting in lower PVAI (0.59 ± 0.23 vs 0.79 ± 0.13 J/m^2^, *p* < 0.001) than HCs. Furthermore, patients with CA had lower WE (76.55 [IQR: 73.05, 81.90] vs 80.90 [IQR: 76.92, 83.35]%, *p* = 0.005) than HCs, indicating impaired energy metabolism. In terms of VAC, patients with CA exhibited higher Ea (1.75 [IQR: 1.40, 2.32] vs 1.45 [IQR: 1.30, 1.80] mmHg/mL, *p* = 0.007), leading to ventricular-arterial decoupling, defined as an increased VAC index (55.17 [IQR: 39.87, 64.21] vs 45.03 [IQR: 36.72, 55.67]%, *p* = 0.014).

### CMR and myocardial work characteristics between AL-CA and ATTR-CA patients

**Fig. 2:**
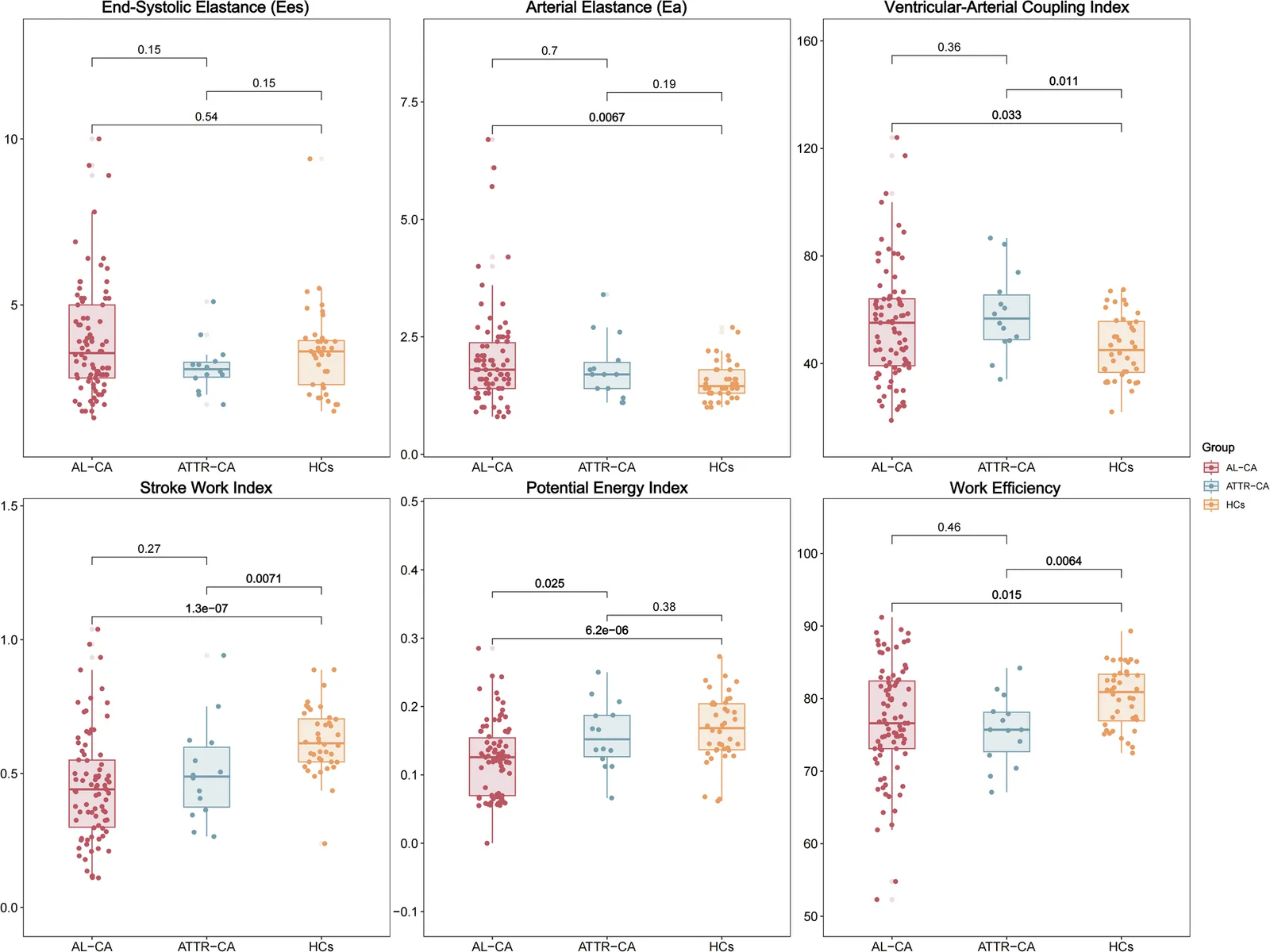
Box plots comparing PV-loop parameters between HCs and patients with light-chain and transthyretin-related amyloidosis

The CMR characteristics of patients with AL-CA and ATTR-CA are summarized in Table 3. There were no statistically significant differences in biventricular cardiac volume and function, LV strain, global native T1, ECV, or T2 values between the two subtypes of patients with CA (all *p* > 0.05). In terms of myocardial work, patients with AL-CA had lower PEI (0.13 [IQR: 0.07, 0.15] vs 0.15 [IQR: 0.13, 0.19] J/m^2^, *p* = 0.025) than patients with ATTR-CA, indicating impaired cardiac reserve function. However, SWI and WE were comparable between the two groups (all *p* > 0.05). Regarding VAC, although both CA subtypes exhibited ventricular-arterial decoupling, there were no significant differences in Ees or Ea between the two groups (all *p* > 0.05). Fig. 2 depicts myocardial work and VAC comparison among patients with AL-CA, ATTR-CA, and HCs.

**Table 3:** CMR and PVLoop characteristics in patients with light-chain and transthyretin-related amyloidosis

	Total CA patients (*n* = 100)	AL-CA (*n* = 86)	ATTR-CA (*n* = 14)	*p* value
CMR parameters				
LV wall thickness (mm)	15.85 ± 3.31	15.85 ± 3.10	15.86 ± 4.54	0.991
LVEDVi (mL/m^2^)	70.20 (62.54, 78.55)	70.06 (62.43, 77.18)	75.78 (64.60, 79.75)	0.343
LVESVi (mL/m^2^)	28.88 ± 7.30	28.63 ± 7.33	30.39 ± 7.24	0.412
LVMassi (g/m^2^)	77.78 (64.63, 95.51)	77.65 (65.44, 93.31)	77.78 (60.83, 106.5)	0.839
LVEF (%)	58.32 (54.22, 64.09)	58.32 (54.85, 64.68)	58.35 (53.47, 62.02)	0.502
RVEDVi (mL/m^2^)	59.91 (50.88, 76.90)	59.05 (50.70, 76.34)	70.34 (54.25, 81.89)	0.358
RVESVi (mL/m^2^)	25.86 (21.40, 34.24)	25.66 (20.84, 34.14)	30.13 (25.29, 37.38)	0.348
RVEF (%)	54.78 ± 9.91	54.45 ± 10.11	56.77 ± 8.64	0.376
LV-GLS (%)	−14.59 ± 4.75	−14.75 ± 4.90	−13.64 ± 3.73	0.338
LV-GCS (%)	−21.36 (−23.66, −18.73)	−21.36 (−23.62, −18.77)	−21.24 (−23.27, −18.11)	0.637
LV-GRS (%)	60.13 (44.91, 75.61)	63.44 (46.46, 77.02)	51.80 (37.75, 57.01)	0.051
Global native T1 (ms)	1421.94 ± 67.54	1424.46 ± 68.09	1406.46 ± 64.24	0.348
Global ECV (%)	46.53 ± 8.54	46.73 ± 8.26	45.28 ± 10.35	0.625
Global native T2 (ms)	45.05 ± 3.33	45.21 ± 3.23	44.04 ± 3.88	0.3
LGE pattern, *n* (%)				0.061
No LGE	33 (33.00)	30 (34.88)	3 (21.43)	…
Subendocardial LGE	44 (44.00)	40 (46.51)	4 (28.57)	…
Transmural LGE	23 (23.00)	16 (18.60)	7 (50.00)	…
PV-loop analysis				
End-systolic Elastance (Ees, mmHg/mL)	3.30 (2.80, 4.60)	3.55 (2.80, 5.00)	3.06 (2.82, 3.27)	0.149
Arterial Elastance (Ea, mmHg/mL)	1.75 (1.40, 2.32)	1.80 (1.40, 2.38)	1.70 (1.40, 1.96)	0.698
Ventricular-Arterial Coupling index (%)	55.17 (39.87, 64.21)	55.17 (39.22, 64.08)	56.74 (48.93, 65.52)	0.363
Stroke Work (J)	0.70 (0.50, 0.90)	0.70 (0.50, 0.90)	0.75 (0.52, 0.90)	0.371
Stroke Work Index (J/m^2^)	0.44 (0.32, 0.56)	0.44 (0.30, 0.55)	0.49 (0.37, 0.60)	0.27
Potential Energy (J)	0.20 (0.10, 0.30)	0.20 (0.10, 0.20)	0.22 (0.20, 0.30)	**0.031**
Potential Energy Index (J/m^2^)	0.13 (0.07, 0.16)	0.13 (0.07, 0.15)	0.15 (0.13, 0.19)	**0.025**
Pressure–volume area (J)	0.90 (0.70, 1.10)	0.90 (0.70, 1.10)	1.00 (0.80, 1.10)	0.225
Pressure–volume area index (J/m^2^)	0.59 ± 0.23	0.57 ± 0.23	0.66 ± 0.21	0.17
Work Efficiency (%)	76.73 ± 7.42	76.91 ± 7.78	75.66 ± 4.78	0.422

*LGE* late gadolinium enhancement, *L/RVEF* left/right ventricular ejection fraction, *LVEDVi/RVEDVi* left/right ventricular end-diastolic volume index, *LVESVi/RVESVi* left/right end-systolic volume index, *LV-GCS* left ventricular global circumferential strain, *LV-GLS* left ventricular global longitudinal strain, *LV-GRS* left ventricular global radial strain, *LVMassi* left ventricular mass index, *SBP* systolic blood pressure, *BMI* body mass index, *BSA* body surface area, *cTnT* cardiac troponin T, *DBP* diastolic blood pressure, *dFLC* serum free light-chain difference, *NT-proBNP* N-terminal pro-B-type natriuretic peptide, *SBP* systolic blood pressure, *SCr* serum creatine

Bold values indicate statistical significance *p* < 0.05

### Correlations of clinical and CMR characteristics in patients with CA

**Fig. 3:**
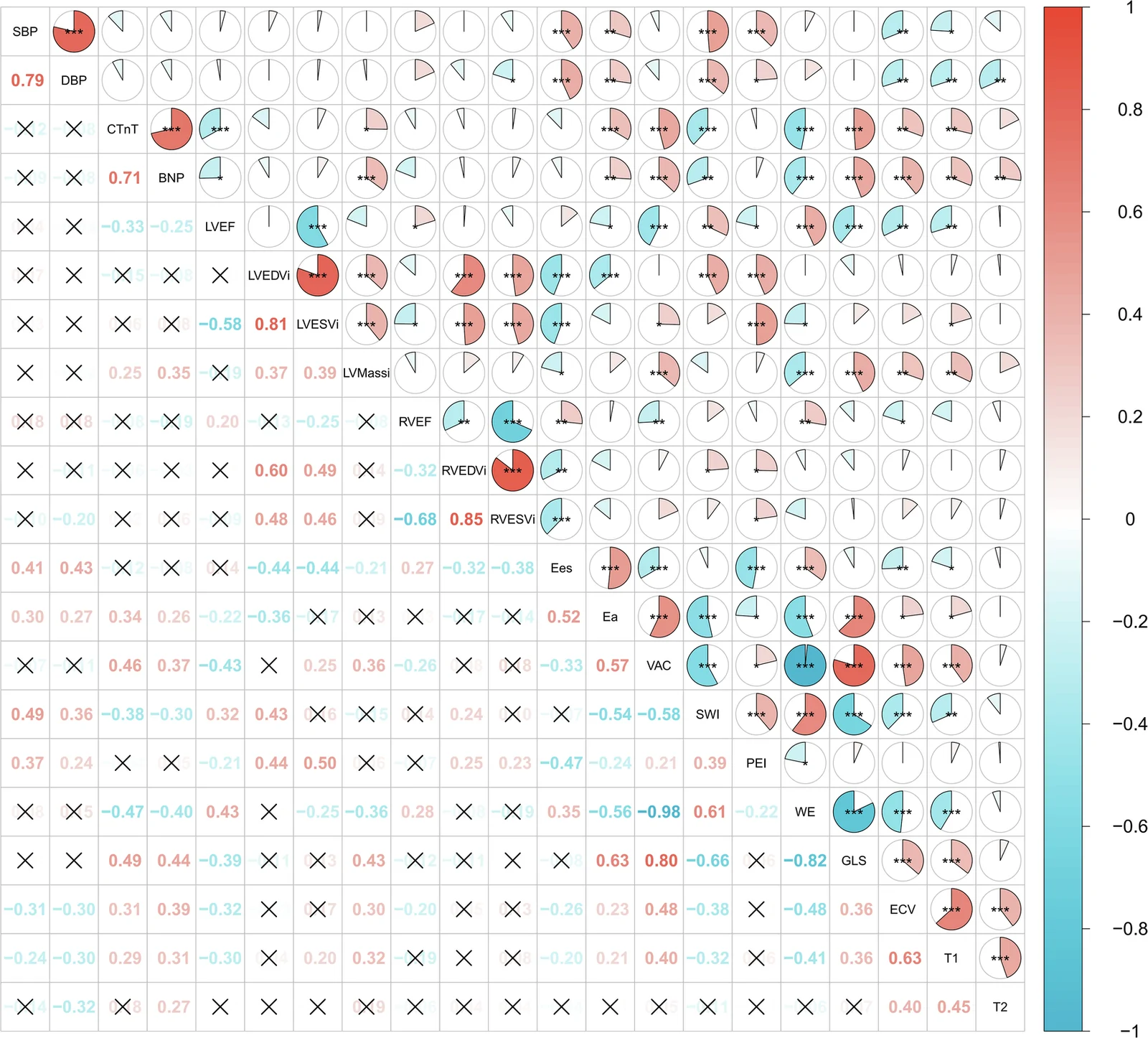
Correlation heatmap of clinical characteristics, cardiac function, myocardial work, and tissue mapping in all patients. L/RVEF, left/right ventricular ejection fraction; LVEDVi/RVEDVi, left/right ventricular end-diastolic volume index; LVESVi/RVESVi, left/right end-systolic volume index; GLS, global longitudinal strain; LVMassi, left ventricular mass index; SBP, systolic blood pressure; DBP, diastolic blood pressure; cTnT, cardiac troponin T; BNP, N-terminal pro-B-type natriuretic peptide; ECV, extracellular volume; Ea, arterial elastance; Ees, end-systolic elastance; SWI, stroke work index; PEI, potential energy index; WE, work efficiency; VAC, ventricular-arterial coupling. ^*^ *p* < 0.05, ^**^ *p* < 0.01, ^***^ *p* < 0.001

The correlations between baseline clinical, laboratory, conventional CMR, and myocardial work parameters are presented in Fig. 3. WE demonstrated moderate-to-strong negative correlations with lgNT-proBNP (*r* = −0.40, *p* < 0.001), lgcTnT (*r* = −0.47, *p* < 0.001), LV-GLS (*r* = −0.82, *p* < 0.001), ECV (*r* = −0.48, *p* < 0.001), and native T1 value (*r* = −0.41, *p* < 0.001), and moderate positive correlations with LVEF (*r* = 0.43, *p* < 0.001). VAC exhibited moderate-to-strong positive correlations with lgNT-proBNP (*r* = 0.46, *p* < 0.001), lgcTnT (*r* = 0.37, *p* < 0.001), LV-GLS (*r* = 0.80, *p* < 0.001), ECV (*r* = 0.48, *p* < 0.001), and native T1 value (*r* = 0.40, *p* < 0.001), and moderate negative correlations with LVEF (*r* = −0.43, *p* < 0.001). Additionally, SBP exhibited mild-to-moderate positive correlations with SWI (*r* = 0.49, *p* < 0.001) and PEI (*r* = 0.37, *p* < 0.001). The correlation coefficient heatmaps for patients with AL-CA and ATTR-CA are provided in Supplemental Fig. 1.

### Diagnostic efficiency of myocardial work in AL-CA from ATTR-CA differentiation

To distinguish AL-CA from ATTR-CA, we constructed a multivariable logistic regression model. Serum NT-proBNP levels (β = −1.94, odds ratio [OR] = 0.14, 95% confidence interval [CI]: 0.03–0.64, *p* = 0.016) and PEI (β = 0.20, OR = 1.23, 95% CI: 1.07–1.44, *p* = 0.007) were finally identified as independent predictors for distinguishing AL-CA from ATTR-CA (Table 4).

**Table 4:** Univariable and multivariable analysis for the differentiation between light-chain and transthyretin-related amyloidosis

	Univariable Analysis			Multivariable Analysis		
	β	Odds ratios (95% CI)	*p* value	β	Odds ratios (95% CI)	*p* value
Lg(cTnT)	−2.20	0.11 (0.02–0.71)	**0.020**	…	…	…
Lg(NT-proBNP)	−1.55	0.21 (0.07–0.61)	**0.004**	−1.94	0.14 (0.03–0.64)	**0.016**
PEI (per 0.01 J/m^2^ increase)	0.11	1.12 (1.01–1.25)	**0.033**	0.20	1.23 (1.07–1.44)	**0.007**

*cTnT* cardiac troponin T, *NT-proBNP* N-terminal pro-B-type natriuretic peptide, *PEI* potential energy index

Bold values indicate statistical significance *p* < 0.05

**Fig. 4:**
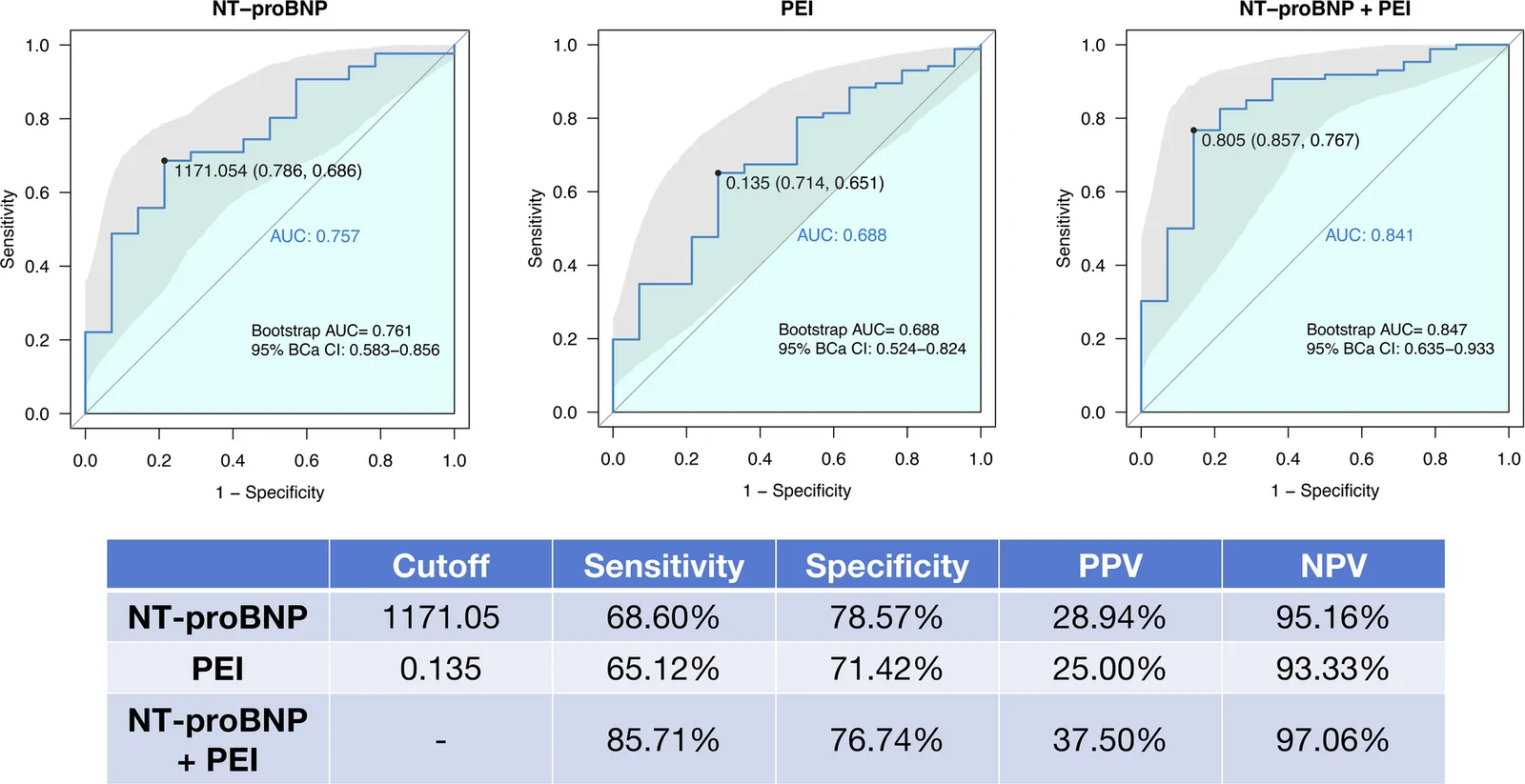
ROC of NT-proBNP and PEI in differentiating light-chain and transthyretin-related amyloidosis patients (Shading around each line is the standard error of the mean computed from 2000 bootstrap resamples. 95% CI for AUC is from 2000 bootstrap draws). Bca, bias-corrected and accelerated; CI, confidence interval; PEI, potential energy index; AUC, area under the receiver operating characteristic curve; NT-proBNP, N-terminal pro-B-type natriuretic peptide; PPV, positive predictive value; NPV, negative predictive value

Figure 4 illustrates the diagnostic accuracy of serum NT-proBNP level and PEI in differentiating AL-CA from ATTR-CA. The optimal single cutoff values, determined by Youden’s index to maximize sensitivity and specificity, were 1171.05 pg/mL for serum NT-proBNP level (area under the curve [AUC] = 0.757, bootstrapping AUC = 0.761, 95% CI: 0.583–0.856) and 0.135 J/m^2^ for PEI (AUC = 0.688, bootstrapping AUC = 0.688, 95% CI: 0.524–0.824). Notably, combining these two parameters enhanced the predictive accuracy, achieving an AUC of 0.841 (bootstrapping AUC = 0.847, 95% CI: 0.635–0.933).

### Reproducibility analysis

Reproducibility results are presented in Supplemental Table 1. The intra-observer and inter-observer reproducibility was excellent for PV-loop parameters derived from the fast long-axis method, with all ICCs greater than 0.85 and relatively low bias. Moreover, PV-loop parameters derived from 2-, 3-, and 4-chamber long-axis cine images showed no significant differences compared to those obtained from 4-chamber images alone (Supplemental Table 2).

## Discussion

This study employed CMR-derived PV loop analysis to systematically compare clinical characteristics and myocardial work between patients with AL-CA and ATTR-CA with preserved LVEF. The findings were as follows: (1) compared to HCs, CA patients with preserved LVEF exhibited significantly lower SBP, greater LV wall thickness and LVMassi, elevated native T1, T2 and ECV values, and impaired PV‑loop parameters; (2) PV‑loop parameters demonstrated correlations with disease activity, cardiac functional indices, and myocardial tissue characteristics, particularly in patients with AL‑CA; (3) patients with AL-CA had higher NT-proBNP and cTnT levels but lower PEI than those of ATTR-CA. A combination of PEI and NT-proBNP enabled the differentiation of AL-CA and ATTR-CA subtypes, suggesting the potential clinical utility of PV-loop parameters for amyloidosis subtyping.

Distinct amyloid fibril subtypes induce markedly different myocardial pathophysiological alterations due to their unique molecular structures and tissue deposition patterns, generating characteristic echocardiographic and CMR imaging findings [29]. Echocardiographic studies have demonstrated that although AL-CA and ATTR-CA share similar echocardiographic features [10, 30], ATTR-CA exhibits a significantly greater prevalence of microcalcification clusters, particularly in the interventricular septum, along with higher echocardiographic pixel intensity than AL-CA [31, 32]. These subtypes demonstrate fundamentally different spatial deposition patterns. CMR studies also showed that ATTR-CA typically exhibits diffuse transmural infiltration, as Dungu et al [33] reported a 90% prevalence of transmural LGE. Conversely, AL-CA primarily exhibited subendocardial accumulation, as Fontana et al [34] identified subendocardial LGE as its diagnostic hallmark. Paradoxically, although patients with ATTR-CA typically present with greater amyloid deposition, AL-CA demonstrates more aggressive clinical progression [35]. Our data corroborated prior reports that AL-CA is associated with significantly elevated NT-proBNP and cTnT levels [36–38]. These findings suggested that proteotoxicity, rather than mechanical infiltration alone, drives disease severity in AL-CA.

Quantitative tissue characterization using CMR mapping techniques provides enhanced assessment of myocardial histological characteristics. Evidence from Baggiano et al [39] indicated that ATTR-CA is associated with higher ECV but lower native T1 values relative to AL-CA. The ECV expansion in ATTR-CA is underpinned by both amyloid infiltration and myocardial hypertrophy, while in AL-CA it is mediated primarily by infiltration alone [40], explaining the more substantial ECV elevation in ATTR-CA. Furthermore, due to the greater direct cardiotoxicity of AL fibrils, which provoke more pronounced myocardial edema than ATTR amyloidosis, native T2 values are typically elevated in AL-CA compared to ATTR-CA [41, 42]. Notably, however, mapping parameters, particularly native T1, are influenced by factors such as disease stage, imaging protocols, and the presence of myocardial edema. As a result, the diagnostic performance of these parameters remains controversial across studies [39–42]. In our study, no significant statistical difference was observed between the CMR mapping parameters. This may be due to the differences in patient populations included in our study. We only included patients with preserved LVEF to achieve early differentiation, whereas previous studies included patients with reduced LVEF [33, 34, 36, 42].

Although LVEF is typically preserved in CA, subclinical myocardial alterations can be detected using strain imaging. In early amyloid deposition, LV-GLS is often the first parameter to decline, primarily due to subendocardial involvement [43]. In contrast, LV-GCS and LV-GRS are more affected by subepicardial or transmural injury, which may serve as a compensatory mechanism for LV-GLS reduction [44]. Furthermore, owing to the direct myocardial toxicity associated with amyloid light-chain deposition in AL-CA and the compensatory ventricular hypertrophy commonly observed in ATTR-CA, previous studies have reported systematically better-preserved cardiac function metrics in ATTR-CA compared with AL-CA [36]. In the present study, although patients with ATTR-CA seemingly had a higher trend of transmural LGE, LV strain parameters did not demonstrate significant discriminative capacity between these two CA subtypes.

Compared with strains, myocardial work assessment provides a more precise differentiation between intrinsic myocardial dysfunction and alterations in myocardial strain resulting from changes in loading conditions [45]. The evaluation of myocardial work was initially performed using PSL derived from echocardiographic studies. Clemmensen et al [46] demonstrated that patients with CA exhibited a significantly reduced LV myocardial work index compared with HCs, with greater disparities emerging during physiological stress, demonstrating impaired myocardial energy utilization. Our results align with these findings, exhibiting reduced WE and elevated VAC index across both subtypes, consistent with cardiac metabolic dysfunction. Palmiero et al [16] observed lower LV myocardial WE in patients with AL-CA than those with ATTR-CA, potentially attributable to circulating free light chain (FLC) cytotoxicity. However, our cohort exhibited no significant subtype differences in WE. This may reflect earlier disease stages in our population, unlike Palmiero et al [16] that included cases with mildly reduced LVEF (40–50%). Interestingly, PEI served as a novel discriminator, demonstrating a significant reduction in patients with AL-CA. Although the addition of PEI to NT-proBNP did not significantly enhance discriminant capacity in this study, it revealed pathophysiological implications, suggesting more severely compromised cardiac reserve function in AL-CA patients [27], potentially attributable to FLC toxicity.

To our knowledge, this is the first exploratory CMR study investigating the pathophysiological and diagnostic implications of PV-loop analysis in AL-CA and ATTR-CA. However, certain limitations must be acknowledged. First, the sample size of patients with ATTR-CA and preserved LVEF in this study was limited (*n* = 14), largely attributable to the current challenges of underdiagnosis in clinical practice [47]. This small cohort size increases the risk of statistical overfitting. Consequently, the optimal cutoff values and differential diagnostic performance of PV-loop parameters identified here require further external validation in larger, independent patient populations. Additionally, while no statistically significant difference was found between PV-loop parameters derived from single-chamber vs multi-chamber analyses, it faces technical challenges in patients with arrhythmias, requiring either tri-plane image acquisition or careful selection of loops with matched RR intervals to ensure reliability. Future studies should explore beat-to-beat averaging algorithms to mitigate arrhythmia-related noise and improve measurement robustness in clinically complex cases. Last but not least, measuring blood pressure due to the position might underestimate the myocardial work in patients with CA, as orthostatic hypotension could occur in some patients in the sitting position [12].

## Conclusion

CMR-derived noninvasive LV PV-loop analysis provides comprehensive evaluation of cardiac performance and thermodynamic efficiency. Patients with AL-CA exhibited higher NT-proBNP levels and lower PEI than those with ATTR-CA. By providing pathophysiological insights into AL-CA and ATTR-CA distinctions, PV-loop parameters have the potential to serve as a complementary, contrast-free approach for differential diagnosis.

## Supplementary information

ELECTRONIC SUPPLEMENTARY MATERIAL

## Data Availability

Data will be made available on reasonable request.

## Abbreviations

AL-CA: Light-chain cardiac amyloidosis
ATTR-CA: Transthyretin-related cardiac amyloidosis
CA: Cardiac amyloidosis
CMR: Cardiovascular magnetic resonance
ECV: Extracellular volume
LGE: Late gadolinium enhancement
LVEF: Left ventricular ejection fraction
LV-GLS: Left ventricular global longitudinal strain
PEI: Potential energy index
PV-loop: Pressure–volume loop
SWI: Stroke work index
VAC: Ventricular-arterial coupling
WE: Work efficiency

## References

[1] Ney S, Ihle P, Ruhnke T et al (2023) Epidemiology of cardiac amyloidosis in Germany: a retrospective analysis from 2009 to 2018. Clin Res Cardiol 112:401–408. 10.1007/s00392-022-02114-y PMC9998316 36241897

[2] Manolis AS, Manolis AA, Manolis TA, Melita H (2019) Cardiac amyloidosis: an underdiagnosed/underappreciated disease. Eur J Intern Med 67:1–13. 10.1016/j.ejim.2019.07.022 31375251

[3] Merlini G, Palladini G (2013) Light chain amyloidosis: the heart of the problem. Haematologica 98:1492–1495. 10.3324/haematol.2013.094482 PMC3789451 24091927

[4] Ruberg FL, Berk JL (2012) Transthyretin (TTR) cardiac amyloidosis. Circulation 126:1286–1300. 10.1161/CIRCULATIONAHA.111.078915 PMC3501197 22949539

[5] Noborn F, Thomsen C, Vorontsov E et al (2023) Subtyping of cardiac amyloidosis by mass spectrometry-based proteomics of endomyocardial biopsies. Amyloid 30:96–108. 10.1080/13506129.2022.2127088 36209425

[6] Fontana M, Ioannou A, Cuddy S et al (2025) The last decade in cardiac amyloidosis: advances in understanding pathophysiology, diagnosis and quantification, prognosis, treatment strategies, and monitoring response. JACC Cardiovasc Imaging 18:478–499. 10.1016/j.jcmg.2024.10.011 PMC12273739 39797879

[7] Jurcuţ R, Onciul S, Adam R et al (2020) Multimodality imaging in cardiac amyloidosis: a primer for cardiologists. Eur Heart J Cardiovasc Imaging 21:833–844. 10.1093/ehjci/jeaa063 32393965

[8] Chatzantonis G, Bietenbeck M, Elsanhoury A et al (2021) Diagnostic value of cardiovascular magnetic resonance in comparison to endomyocardial biopsy in cardiac amyloidosis: a multi-centre study. Clin Res Cardiol 110:555–568. 10.1007/s00392-020-01771-1 PMC8055632 33170349

[9] Steen H, Giusca S, Montenbruck M et al (2021) Left and right ventricular strain using fast strain-encoded cardiovascular magnetic resonance for the diagnostic classification of patients with chronic non-ischemic heart failure due to dilated, hypertrophic cardiomyopathy or cardiac amyloidosis. J Cardiovasc Magn Reson 23:45. 10.1186/s12968-021-00711-w PMC8025329 33823860

[10] Liang S, Liu Z, Li Q, He W, Huang H (2023) Advance of echocardiography in cardiac amyloidosis. Heart Fail Rev 28:1345–1356. 10.1007/s10741-023-10332-3 PMC10575814 37558934

[11] Steen H, Montenbruck M, Kallifatidis A et al (2024) Multi-parametric non-contrast cardiac magnetic resonance for the differentiation between cardiac amyloidosis and hypertrophic cardiomyopathy. Clin Res Cardiol 113:469–480. 10.1007/s00392-023-02348-4 38095711

[12] Liang S, Wan K, Li J, Chen J, Chen Y (2024) Patients with cardiac amyloidosis are more prone to hypotension. J Am Coll Cardiol 84:e233. 10.1016/j.jacc.2024.04.072 39357945

[13] Bastos MB, Burkhoff D, Maly J et al (2020) Invasive left ventricle pressure–volume analysis: overview and practical clinical implications. Eur Heart J 41:1286–1297. 10.1093/eurheartj/ehz552 PMC7084193 31435675

[14] Burkhoff D (1990) The conductance method of left ventricular volume estimation. Methodologic limitations put into perspective. Circulation 81:703–706. 10.1161/01.cir.81.2.703 2404635

[15] Seemann F, Arvidsson P, Nordlund D et al (2019) Noninvasive quantification of pressure–volume loops from brachial pressure and cardiovascular magnetic resonance. Circ Cardiovasc Imaging 12:e008493. 10.1161/CIRCIMAGING.118.008493 30630347

[16] Palmiero G, Rubino M, Monda E et al (2021) Global left ventricular myocardial work efficiency in heart failure patients with cardiac amyloidosis: pathophysiological implications and role in differential diagnosis. J Cardiovasc Echogr 31:157–164. 10.4103/jcecho.jcecho_16_21 PMC8603776 34900551

[17] Gertz MA, Comenzo R, Falk RH et al (2005) Definition of organ involvement and treatment response in immunoglobulin light chain amyloidosis (AL): a consensus opinion from the 10th International Symposium on Amyloid and Amyloidosis, Tours, France, 18–22 April 2004. Am J Hematol 79:319–328. 10.1002/ajh.20381 16044444

[18] Garcia-Pavia P, Rapezzi C, Adler Y et al (2021) Diagnosis and treatment of cardiac amyloidosis: a position statement of the ESC Working Group on Myocardial and Pericardial Diseases. Eur Heart J 42:1554–1568. 10.1093/eurheartj/ehab072 PMC8060056 33825853

[19] Writing Committee, Kittleson MM, Ruberg FL et al (2023) 2023 ACC expert consensus decision pathway on comprehensive multidisciplinary care for the patient with cardiac amyloidosis: a report of the American College of Cardiology Solution Set Oversight Committee. J Am Coll Cardiol 81:1076–1126. 10.1016/j.jacc.2022.11.022. 36697326

[20] Kittleson MM, Panjrath GS, Amancherla K et al (2023) 2023 ACC expert consensus decision pathway on management of heart failure with preserved ejection fraction: a report of the American College of Cardiology Solution Set Oversight Committee. J Am Coll Cardiol 81:1835–1878. 10.1016/j.jacc.2023.03.393 37137593

[21] Wan K, Sun J, Yang D et al (2018) Left ventricular myocardial deformation on cine MR images: relationship to severity of disease and prognosis in light-chain amyloidosis. Radiology 288:73–80. 10.1148/radiol.2018172435 29664336

[22] The Criteria Committee of the New York Heart Association (1994) Nomenclature and criteria for diagnosis of diseases of the heart and great vessels, 9th revised edn. Little, Brown & Co., Boston, pp 253–256

[23] Xu Z, Li W, Wang J et al (2023) Reference ranges of myocardial T1 and T2 mapping in healthy Chinese adults: a multicenter 3T cardiovascular magnetic resonance study. J Cardiovasc Magn Reson 25:64. 10.1186/s12968-023-00974-5 PMC10652608 37968645

[24] McEvoy JW, McCarthy CP, Bruno RM et al (2024) 2024 ESC guidelines for the management of elevated blood pressure and hypertension. Eur Heart J 45:3912–4018. 10.1093/eurheartj/ehae178 39210715

[25] Writing Committee Members, Jones DW, Ferdinand KC et al (2025) 2025 AHA/ACC/AANP/AAPA/ABC/ACCP/ACPM/AGS/AMA/ASPC/NMA/PCNA/SGIM guideline for the prevention, detection, evaluation and management of high blood pressure in adults: a report of the American College of Cardiology/American Heart Association Joint Committee on Clinical Practice Guidelines. Hypertension 82:e212–e316. 10.1161/HYP.0000000000000249. 40811516

[26] Schulz-Menger J, Bluemke DA, Bremerich J et al (2020) Standardized image interpretation and post-processing in cardiovascular magnetic resonance—2020 update: Society for Cardiovascular Magnetic Resonance (SCMR): board of trustees task force on standardized post-processing. J Cardiovasc Magn Reson 22:19. 10.1186/s12968-020-00610-6 PMC7066763 32160925

[27] Liang S, Wan K, Deng Q et al (2026) Characteristics and clinical significance of myocardial work in cardiac light-chain amyloidosis: pressure–volume loop analysis based on cardiovascular magnetic resonance. J Cardiovasc Magn Reson 28:102716. 10.1016/j.jocmr.2026.102716 PMC13319382 41861917

[28] Youden WJ (1950) Index for rating diagnostic tests. Cancer 3:32–35. 10.1002/1097-0142(1950)3:1<32::aid-cncr2820030106>3.0.co;2-3 15405679

[29] Liang S, Huang H, Chen Y (2025) Echocardiography in cardiac amyloidosis: from identification to classification. JACC Asia 5:647–649. 10.1016/j.jacasi.2025.03.002 PMC12081234 40266181

[30] Liang S, Liu Z, Shi F et al (2025) Development and validation of an echocardiographic nomogram for identifying cardiac amyloidosis in patients with left ventricular hypertrophy. BMC Cardiovasc Disord 25:767. 10.1186/s12872-025-04973-5 PMC12557862 41146005

[31] Slostad B, Appadurai V, Narang A et al (2024) Novel echocardiographic pixel intensity quantification method for differentiating transthyretin cardiac amyloidosis from light chain cardiac amyloidosis and other phenocopies. Eur Heart J Cardiovasc Imaging 25:1601–1611. 10.1093/ehjci/jeae095 38581424

[32] Sugimoto T, Kayama K, Kawada Y et al (2025) Relative interventricular septal brightness for subtype diagnosis of cardiac amyloidosis: results from the JSE J-CASE study. Int J Cardiol 429:133164. 10.1016/j.ijcard.2025.133164 40088957

[33] Dungu JN, Valencia O, Pinney JH et al (2014) CMR-based differentiation of AL and ATTR cardiac amyloidosis. JACC Cardiovasc Imaging 7:133–142. 10.1016/j.jcmg.2013.08.015 24412186

[34] Fontana M, Pica S, Reant P et al (2015) Prognostic value of late gadolinium enhancement cardiovascular magnetic resonance in cardiac amyloidosis. Circulation 132:1570–1579. 10.1161/CIRCULATIONAHA.115.016567 PMC4606985 26362631

[35] Schilling JD, Nuvolone M, Merlini G (2024) The pathophysiological and therapeutic implications of cardiac light-chain amyloidosis compared with transthyretin amyloidosis. JACC Heart Fail 12:1781–1787. 10.1016/j.jchf.2024.07.003 39177569

[36] Neculae G, Adam R, Jercan A et al (2024) Cardiac amyloidosis is not a single disease: a multiparametric comparison between the light chain and transthyretin forms. ESC Heart Fail 11:2825–2834. 10.1002/ehf2.14852 PMC11424370 38757395

[37] Rapezzi C, Merlini G, Quarta CC et al (2009) Systemic cardiac amyloidoses: disease profiles and clinical courses of the 3 main types. Circulation 120:1203–1212. 10.1161/CIRCULATIONAHA.108.843334 19752327

[38] Suhr OB, Anan I, Backman C et al (2008) Do troponin and B-natriuretic peptide detect cardiomyopathy in transthyretin amyloidosis? J Intern Med 263:294–301. 10.1111/j.1365-2796.2007.01888.x 18069997

[39] Baggiano A, Boldrini M, Martinez-Naharro A et al (2020) Noncontrast magnetic resonance for the diagnosis of cardiac amyloidosis. JACC Cardiovasc Imaging 13:69–80. 10.1016/j.jcmg.2019.03.026 31202744

[40] Fontana M, Banypersad SM, Treibel TA et al (2015) Differential myocyte responses in patients with cardiac transthyretin amyloidosis and light-chain amyloidosis: a cardiac MR imaging study. Radiology 277:388–397. 10.1148/radiol.2015141744 25997029

[41] Ridouani F, Damy T, Tacher V et al (2018) Myocardial native T2 measurement to differentiate light-chain and transthyretin cardiac amyloidosis and assess prognosis. J Cardiovasc Magn Reson 20:58. 10.1186/s12968-018-0478-3 PMC6097442 30115079

[42] Kravchenko D, Isaak A, Zimmer S et al (2024) Parametric mapping using cardiovascular magnetic resonance for the differentiation of light chain amyloidosis and transthyretin-related amyloidosis. Eur Heart J Cardiovasc Imaging 25:1451–1461. 10.1093/ehjci/jeae154 38912832

[43] Claus P, Omar AMS, Pedrizzetti G, Sengupta PP, Nagel E (2015) Tissue tracking technology for assessing cardiac mechanics: principles, normal values, and clinical applications. JACC Cardiovasc Imaging 8:1444–1460. 10.1016/j.jcmg.2015.11.001 26699113

[44] Slivnick JA, Singulane C, Sun D et al (2023) Preservation of circumferential and radial left ventricular function as a mitigating mechanism for impaired longitudinal strain in early cardiac amyloidosis. J Am Soc Echocardiogr 36:1290–1301. 10.1016/j.echo.2023.08.005 37574149

[45] Meng F, Li J, Zhao R et al (2025) Myocardial work indices in the short-term prognosis of light-chain cardiac amyloidosis: incremental value beyond traditional staging models and echocardiographic parameters. Br J Haematol 207:1962–1972. 10.1111/bjh.70122 40967888

[46] Clemmensen TS, Eiskjær H, Mikkelsen F et al (2020) Left ventricular pressure-strain-derived myocardial work at rest and during exercise in patients with cardiac amyloidosis. J Am Soc Echocardiogr 33:573–582. 10.1016/j.echo.2019.11.018 32061410

[47] Aimo A, Vergaro G, Castiglione V et al (2024) Wild-type transthyretin cardiac amyloidosis is not rare in elderly subjects: the CATCH screening study. Eur J Prev Cardiol 31:1410–1417. 10.1093/eurjpc/zwae093 38456769

