# Supplementary material for: Myocardial work in light-chain and transthyretin-related amyloidosis with preserved ejection fraction: a cardiovascular magnetic resonance study
Source: Insights Imaging. 2026 Oct 9;17:245. doi: 10.1186/s13244-026-02408-z (PMC13653009; doi:10.1186/s13244-026-02408-z)
Supplement: Supplementary file 1 [file 13244_2026_2408_MOESM1_ESM.pdf]

Myocardial Work in Light-Chain and Transthyretin-Related Amyloidosis with Preserved Ejection Fraction: A Cardiovascular Magnetic Resonance Study

ELECTRONIC SUPPLEMENTARY MATERIAL

Supplemental Methods

Diagnostic criteria

AL amyloidosis was confirmed by the presence of monoclonal plasma cell dyscrasia and positive immunohistochemical staining or mass spectrometry for κ or λ light chains in the biopsy specimen [1]. ATTR amyloidosis was confirmed by biopsy with positive mass spectrometry, immunohistochemistry identification, or ^{99m}Tc-PYP scintigraphy [2,3]. TTR gene sequencing was performed to distinguish hereditary from wild-type ATTR.

Details of the included Patients

Among the 86 patients with AL-CA, all diagnoses were biopsy-confirmed. The biopsy sites were distributed as follows: Kidney (28 cases, 32.56%), abdominal fat (28 cases, 32.56%), bone marrow (18 cases, 20.93%), endomyocardium (7 cases, 8.14%), liver (2 cases, 2.33%), tongue and labial gland (2 cases, 2.33%), and gastrointestinal tract (1 case, 1.16%). Among the 14 patients with ATTR-CA, 7 were biopsy-confirmed, and the remaining 7 were diagnosed using noninvasive imaging modalities. The biopsy sites included endomyocardium (3 cases, 42.86%), eyes (2 cases, 28.57%), kidneys (1 case, 14.29%), and abdominal fat (1 case, 14.29%). Among all patients with ATTR-CA, 1 had wild-type (7.14%), and 13 had hereditary (92.86%). The specific amino acid mutations identified were Ala117Ser (3 cases, 23.08%), Phe64Ser (2 cases, 15.38%), Glu74Gln (1 case, 7.69%), Thr80Ala (1 case, 7.69%), Tyr89His (1 case, 7.69%), Gly73Ala (1 case, 7.69%), Gly67Glu (1 case, 7.69%), Val50Leu (1 case, 7.69%), Ala45Thr (1 case, 7.69%), and Ile104Asn (1 case, 7.69%).

Cardiac MR Imaging Protocol

CMR imaging was performed on a 3T scanner (MAGNETOM Trio or Skyra, Siemens Healthcare, Erlangen, Germany) using a dedicated 32-channel phased-array cardiac coil. Imaging was conducted with breath-holding techniques and electrocardiographic gating to ensure image quality. Steady-state free precession (SSFP) cine images were acquired in continuous short-axis and horizontal long-axis orientations. The typical scan parameters were as follows: repetition time (TR) of 3.4 milliseconds, echo time (TE) of 1.3 milliseconds, flip angle of 50°, field of view (FOV) ranging from 320 to 340 mm², matrix size of 256×144, and slice thickness of 8 mm with no gap.T1 mapping was performed in three short-axis and 4-chamber views using the Modified Look-Locker Inversion recovery (MOLLI) sequence with both a 5b(3b)3b and a 4b(1b)3b(1b)2b scheme before and 10-15 minutes after gadolinium injection (where b indicates heart beat). The typical scan parameters for T1 mapping were as follows: TR, 300 ms; TE, 1.2 ms; flip angle, 35°; FOV, 270×320 mm²; matrix size, 144×256; slice thickness, 8 mm. The typical scan parameters for T2 mapping were: TR, 240 ms; TE, 1.0 ms; flip angle, 12°; FOV, 320×340 mm²; matrix size, 114×176; slice thickness, 8 mm; T2 preparation pulse with spin echo times of 0, 30, and 55 ms.

LGE image acquisition was initiated simultaneously with the bolus administration of 0.1 mL/kg of a gadolinium-based contrast agent (Magnevist, Bayer Schering Pharma) at an infusion rate of 3.0 mL/sec. The imaging parameters for first - pass perfusion were: FOV, 270×360 mm²; flip angle, 10°; matrix size, 200×280; slice thickness, 8 mm; TR, 153.8 msec; TE, 1.06 msec; bandwidth, 1184 Hz/pixel; 80 dynamics. LGE images were obtained in consecutive short - axis and horizontal long-axis views using a phase-sensitive inversion recovery (PSIR) sequence 10 - 15 minutes post-injection of gadolinium-based contrast (0.15 mmol/kg/bolus). The sequence parameters for LGE imaging were: TR, 700 msec; TE, 1.56 msec; flip angle, 20°; matrix size, 256×144.

CMR Post-processing and Analysis for T1 Mapping

Native T1 and ECV of the myocardium were quantified on the same basal and mid-ventricular short-axis slices using QMap package, version 2.2.6 (Medis Medical Imaging Systems, Leiden, The Netherlands). The myocardial region of interest (ROI) was carefully contoured on end-diastolic images, with meticulous attention to the endocardial and

epicardial borders to exclude endocardial trabeculations, blood pool, and epicardial fat. Native T1 and T2 values were derived from motion-corrected images by fitting an exponential decay curve to each pixel within the ROIs. ECV was calculated using the formula: $ECV = (1 - hematocrit) \times (1/T1_{myo\text{-}post} - 1/T1_{myo\text{-}pre}) / (1/T1_{blood\text{-}post} - 1/T1_{blood\text{-}pre})$ [4]. Hematocrit levels were measured on the same day as the CMR examination for healthy controls and within a 3-day window for patients. Special precautions were taken to exclude image artifacts from myocardial measurements to ensure data accuracy and reliability. Global native T1, T2 and ECV values were determined as the mean of measurements obtained from the basal and mid-ventricular short-axis slices [5].

References

1. Gertz MA, Comenzo R, Falk RH, et al (2025) Definition of organ involvement and treatment response in immunoglobulin light chain amyloidosis (AL): a consensus opinion from the 10th International Symposium on Amyloid and Amyloidosis, Tours, France, 18-22 April 2004. Am J Hematol 79(4):319-328. doi: 10.1002/ajh.20381
2. Garcia-Pavia P, Rapezzi C, Adler Y, et al (2021) Diagnosis and treatment of cardiac amyloidosis: a position statement of the ESC Working Group on Myocardial and Pericardial Diseases. Eur Heart J 42(16):1554-1568. doi: 10.1093/eurheartj/ehab072
3. Writing Committee, Kittleson MM, Ruberg FL, et al (2023) 2023 ACC Expert Consensus Decision Pathway on Comprehensive Multidisciplinary Care for the Patient With Cardiac Amyloidosis: A Report of the American College of Cardiology Solution Set Oversight Committee. J Am Coll Cardiol 81(11):1076-1126. doi: 10.1016/j.jacc.2022.11.022
4. Mongeon FP, Jerosch-Herold M, Coelho-Filho OR, Blankstein R, Falk RH, Kwong RY. Quantification of extracellular matrix expansion by CMR in infiltrative heart disease. JACC Cardiovasc Imaging. 2012;5(9):897-907. doi:10.1016/j.jcmg.2012.04.006
5. Messroghli DR, Moon JC, Ferreira VM, et al. Clinical recommendations for cardiovascular magnetic resonance mapping of T1, T2, T2* and extracellular volume: A consensus statement by the Society for Cardiovascular Magnetic

Resonance (SCMR) endorsed by the European Association for Cardiovascular Imaging (EACVI). J Cardiovasc Magn Reson. 2017;19(1):75. doi:10.1186/s12968-017-0389-8

Supplemental Table 1. Intra- and Inter-observer reproducibility for pressure-volume loop parameters.

	Intraobserver		Interobserver	
	**ICC (95%CI)**	**Bias (Limits of agreement)**	**ICC (95%CI)**	**Bias (Limits of agreement)**
**End-Systolic Elastance (Ees, mmHg/mL)**	0.987 (0.968 ~ 0.995)	-0.356 ~ 0.346	0.950 (0.873 ~ 0.980)	-0.601 ~ 0.751
**Arterial Elastance (mmHg/mL, Ea)**	0.991 (0.978 ~ 0.997)	-0.153 ~ 0.203	0.905 (0.760 ~ 0.962)	-0.462 ~ 0.682
**Ventricular-Arterial Coupling Index (%)**	0.943 (0.856 ~ 0.977)	-14.174 ~ 19.237	0.924 (0.808 ~ 0.970)	-15.553 ~ 24.005
**Stroke Work (J)**	0.993 (0.982 ~ 0.997)	-0.179 ~ 0.169	0.994 (0.985 ~ 0.998)	-0.174 ~ 0.144
**Potential Energy (J)**	0.918 (0.793 ~ 0.968)	-0.116 ~ 0.156	0.887 (0.716 ~ 0.955)	-0.144 ~ 0.164
**Work Efficiency (%)**	0.984 (0.959 ~ 0.994)	-6.420 ~ 5.350	0.975 (0.938 ~ 0.990)	-4.924 ~ 4.614

ICC, intraclass correlation coefficient.

Supplemental Table 2. Reproducibility for pressure-volume loop parameters results between using 2ch, 3ch, 4ch long-axis cine images and 4ch images only.

	2ch, 3ch, 4ch long-axis cine images (n = 20)	4ch images only (n = 20)	*P* value
**End-Systolic Elastance (Ees, mmHg/mL)**	3.31±1.14	3.42±1.15	0.752
**Arterial Elastance (mmHg/mL, Ea)**	1.8 (1.48, 2.32)	1.75 (1.37, 2.4)	1
**Ventricular-Arterial Coupling Index (%)**	59.38±13.79	57.48±13.25	0.659
**Stroke Work (J)**	0.7 (0.4, 0.8)	0.6 (0.4, 0.83)	1
**Potential Energy (J)**	0.2 (0.2, 0.2)	0.2 (0.18, 0.3)	0.761
**Work Efficiency (%)**	75.31 ± 4.68	75.67 ± 4.84	0.812

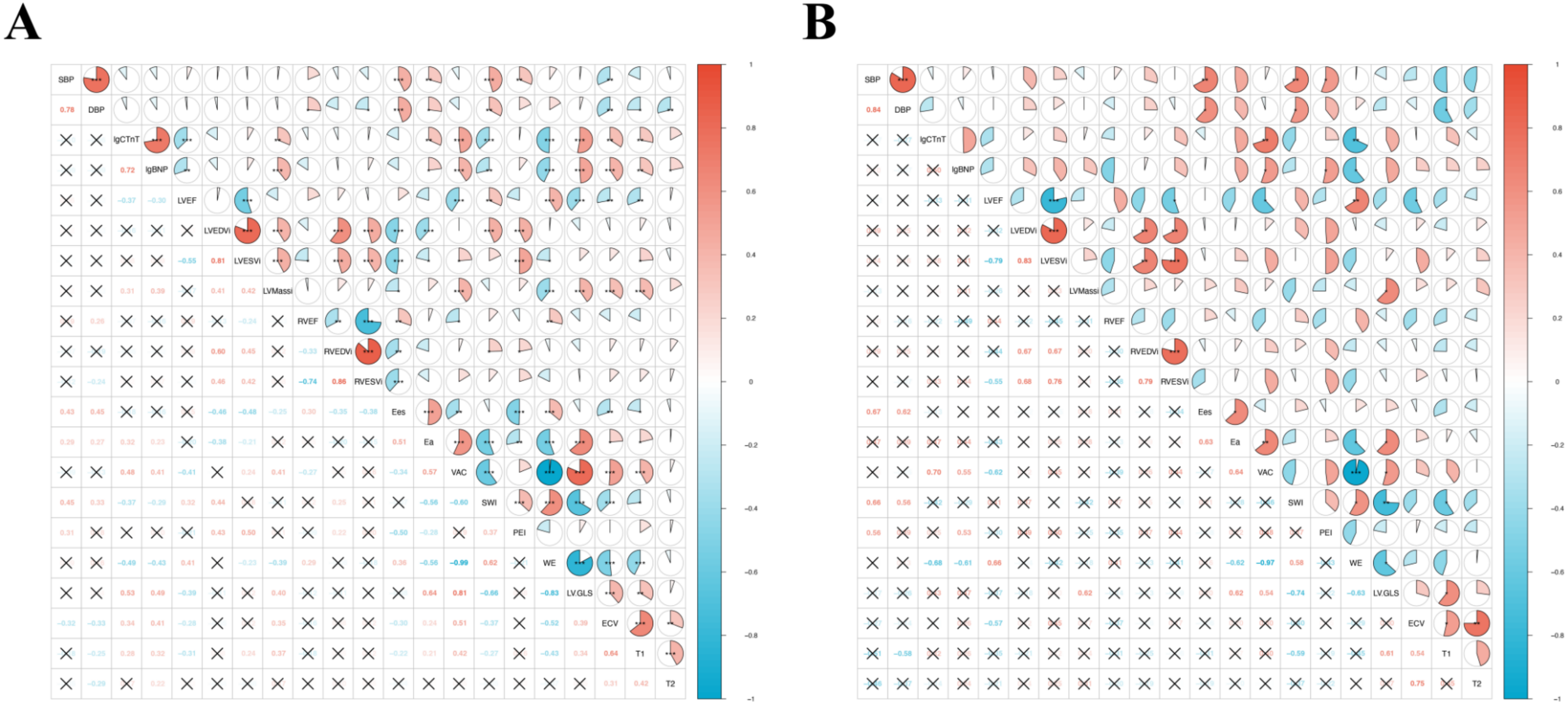

Supplemental Figure 1. Correlation heat map of clinical characters, cardiac function, myocardial word and tissue mapping in (A) AL patients; (B) ATTR patients.
